# Validated methodology for quantifying infestation levels of dreissenid mussels in environmental DNA (eDNA) samples

**DOI:** 10.1038/srep39067

**Published:** 2016-12-14

**Authors:** Luis Peñarrubia, Carles Alcaraz, Abraham bij de Vaate, Nuria Sanz, Carles Pla, Oriol Vidal, Jordi Viñas

**Affiliations:** 1 Laboratori d’Ictiologia Genètica, Department of Biology, Universitat de Girona, LEAR Building, Girona, 17003, Spain; 2 IRTA Aquatic Ecosystems, Sant Carles de la Ràpita, 43540, Spain; 3 Waterfauna Hydrobiologisch Adviesbureau, Oostrandpark 30, 8212 AP Lelystad, The Netherlands

## Abstract

The zebra mussel (*Dreissena polymorpha* Pallas, 1771) and the quagga mussel (*D. rostriformis* Deshayes, 1838) are successful invasive bivalves with substantial ecological and economic impacts in freshwater systems once they become established. Since their eradication is extremely difficult, their detection at an early stage is crucial to prevent spread. In this study, we optimized and validated a qPCR detection method based on the histone H2B gene to quantify combined infestation levels of zebra and quagga mussels in environmental DNA samples. Our results show specific dreissenid DNA present in filtered water samples for which microscopic diagnostic identification for larvae failed. Monitoring a large number of locations for invasive dreissenid species based on a highly specific environmental DNA qPCR assay may prove to be an essential tool for management and control plans focused on prevention of establishment of dreissenid mussels in new locations.

The zebra mussel, *Dreissena polymorpha* (Pallas, 1771) is a successful invasive bivalve native to the brackish estuaries and freshwaters systems of the Ponto-Caspian regions (Black, Caspian, and Azov Seas). It is considered one of the 100 world’s worst invasive alien species (IUCN-ISSG)^1^ possessing several biological life cycle features that favor its proliferation: rapid growth, early sexual maturity, and high fecundity of up to 1 million eggs per season^2^,^3^. Due to a planktonic larval stage and also facilitated by human-mediated activities such as larvae in ballast water^4^, attachment of adults to hulls of commercial and recreational ships^5^,^6^,^7^.

The zebra mussel was first time found on the Iberian Peninsula in the Ribarroja Reservoir (Ebro River) in 2001^8^, about 100 years later than its first report in Western Europe^2^. This delayed invasion might have been due the Pyrenees mountain range serving as a barrier^9^,^10^ to dispersal. Since then, this species has expanded along the Ebro River and adjacent basins in the northeast region of the Iberian Peninsula^11^,^12^. Moreover, these effects are expected to increase with the imminent arrival of another invasive dreissenid, the quagga mussel (*D. rostriformis* Deshayes, 1838)^10^,^13^,^14^. Around 2004 the quagga mussel arrived in Western Europe^15^,^16^,^17^ and has been constantly spreading into areas where zebra mussels previously invaded^18^,^19^,^20^, and thus are predicted to arrive to the Iberian Peninsula in a near future unless effective prevention measures are enacted^10^.

To face these challenges, the National Strategy for the Control of Zebra Mussel in Spain has focused efforts on the rapid detection of both species^21^. Plans for control and management^22^,^23^ were developed to prevent further zebra mussel expansion since eradication of dreissenids is extremely difficult once they are established^4^,^24^. Therefore it is critical to detect their presence as early as possible, when measures of eradication could be effective^25^,^26^.

The current methodology for detecting the presence of the zebra mussel is rather rudimentary. Adult individual detection is based on visual diagnostics, and veliger larvae are detected using microscopy^25^. The latter is labour intensive and time-consuming, and can result in false positives due to the similar appearance between dreissenid larvae and those of other macroinvertebrates^27^. Alternatively, molecular markers have proven useful in species identification diagnostics, but these previous studies do not intend sample quantification^4^,^28^,^29^. Thus, after optimization they offer a faster diagnosis without false positives but they do not allow the quantification of the dreissenid infestation. The combination of environmental DNA (eDNA) sampling is with a quantitative PCR (qPCR) method^30^,^31^ may be optimized to permit quantification, as evaluated here.

In the present study, we developed and optimized a qPCR procedure to assess the level of dreissenid infestation using eDNA samples. This method is based on a single-copy gene used as a genetic marker from Massive Parallel Sequencing (MPS) output of zebra mussel genomic DNA^11^. This method could be applied to diagnose the dreissenid infestation level in locations at risk of invasion. Evaluation and quantification of these locations will be crucial to complete the management and control plans to prevent future dreissenid spread.

## Results

### Stage A: single-copy gene marker selection

#### Gene annotation, single-copy gene selection and PCR specificity

Analysis of MPS output^11^ using Blast2GO software resulted in a total of 220 Gene Ontology (GO) terms that were tagged on 56 contigs (1.44% of the total 2,326) (see Supplementary Fig. S1). When single-copy genes were screened in contigs larger than 500 bp, just four were found in the BlastX analysis (Table 1). Of these genes, one corresponded to the methionyl-tRNA synthetase (MARS) gene with an E-value of 7.52E-23. The remaining three genes were different histone genes as follows: histone 1 (H1) with an E-value of 1.08E-27; histone 2B (H2B) with 5.31E-54; and histone 3 (H3) with having 3.77E-88 (Table 1). All four markers were verified for species specificity, and three (H1, H2B and MARS) presented a single and clear PCR amplicon for the two dreissenid species (see Supplementary Fig. S2).

#### Standard qPCR curve amplification

Following the species specificity test, we considered that three markers (H1, H2B and MARS) were suitable to be optimized for dreissenid DNA quantification by qPCR. After several attempts, only H1 and H2B markers had a dynamic range with a coefficient of variation (CV) below 0.3 in all concentrations tested (from 50 to 5E-3 ng/μL), characterized by a single peak in the melting curve (Table 1). H2B was selected due to real time amplification efficiency (E = 1.973), lower Blast E-value, and lower coefficient of variation among replicates (CV = 0.154). Adding the concentrations needed for the standard curve, the H2B gene showed both detection level (DL) and quantification level (QL) at 5E-4 ng/μL. In all cases, the sequence of the PCR products matched the reference sequence with a variation of 5 SNPs between the two dreissenid species (see Supplementary Fig. S3).

### Stage B: Environmental quantification

#### PCR amplification

**Figure 1:**
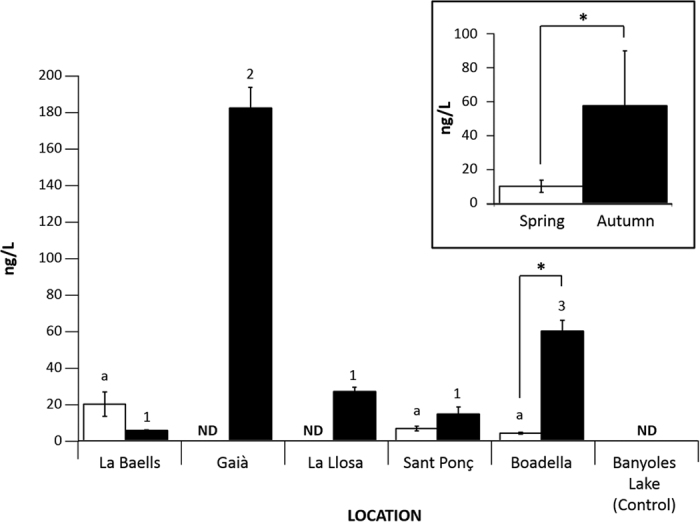
DNA quantification level for each location. White and black bars correspond to spring and autumn sampling periods respectively. Right square represents the total quantification average among all locations for both sampling periods. Pairwise T-Student comparisons between sampling periods significance is represented by asterisks (*P < 0.05). Letters and numbers correspond to statistical identity among spring and autumn comparisons respectively after ANOVA and subsequent Bonferroni post-hoc analysis. ND = non-detected.

The Banyoles Lake eDNA sample confirmed the absence of dreissenid DNA resulting in negative PCR amplification. Positive dreissenid DNA presence was observed in the remaining five locations in at least one of the two temporal samples (Table 2, Fig. 1). In the spring sampling, three locations (La Baells, Sant Ponç and Boadella) were positive for dreissenid DNA, and all five locations were positive in the autumn sampling (Table 2, Fig. 1).

#### Infestation DNA level quantification

As a general pattern, the DNA levels were approximately six times higher (*t* = −2.497; *df* = 14*; P* = 0.029; inset in Fig. 1) in autumn (57.928 ng/L; SEM = 32.591) than in spring (10.151 ng/L; SEM = 3.835). With the exception of La Baells Reservoir, all reservoirs followed this general pattern but only the Boadella Reservoir (*t* = −9.187; *df* = 2; *P* = 0.012) presented statistical significant higher DNA values in autumn samples (Fig. 1). In contrast, La Baells Reservoir presented an opposite pattern with more DNA quantity for the spring season although non-significant differences were detected. The spring season comparison among the three reservoirs with positive dreissenid DNA presence resulted in non-significant differences. In contrast, the autumn season was more variable (*F* = 140.377; *df* = 9*; P* = 0.000) with three significantly differentiated groups: Gaia presented the highest quantification level, Boadella Reservoir in an intermediate position and La Baells, La LLosa and Sant Ponç Reservoirs with the lowest and similar quantification levels (Fig. 1).

## Discussion

Several previous studies have developed detection methods based on molecular markers for dreissenid identification in environmental samples^4^,^28^,^29^. However, to our knowledge, this study is the first published in which a method was developed for identification and quantification of the infestation level of invasive dreissenid species. To achieve these results, we generated and validated a highly specific marker using qPCR quantification of the H2B nuclear single-copy gene. The selection of the maker (H2B single-copy gene) was realized by bioinformatics analyses of a previous MPS published output^11^ with a laboratory validation of the usefulness of the marker. The choice of the marker was based on the high specificity for dreissenid species, which allows the discrimination of dreissenids species from endemic mollusk species. The marker also presented the best qPCR parameters compared to other putative markers (Table 1). Several other studies have targeted mitochondrial DNA based markers for species identification in environmental samples^32^,^33^,^34^ instead of using single copy nuclear genes. The use of mitochondrial DNA is extremely useful for detecting the DNA of dreissenids in locations where the presence is unknown, or where very small numbers are present such as the initial stages of invasion, but nuclear markers are best suited to compare sites with known infestations and quantification^35^,^36^.

Our qPCR methodology developed is not designed to provide the absolute number of larvae or adults in the water body, but it quantifies the amount of dreissenid DNA, which very likely informs about the levels of infestation. As we will discuss, the fact that we were able to detect significant differences in seasonal fluctuations in dreissenid populations strongly supports this assessment. In this context, any source of further variation should be avoided. Thus, although the total amount of DNA (both nuclear and mitochondrial) may vary by several orders of magnitude during ontogeny^27^,^28^,^30^, the number of mtDNA molecules also varies from cell to cell. The use of mtDNA markers is therefore not recommended as they would incorporate additional errors in quantifying the infestation^36^.

This protocol outperforms an alternative quantification using the difference in the number of endpoint PCR replicates with positive detections, even in samples with low DNA presence^37^. Replicative endpoint PCR would experience the same problem of quantifying DNA instead of assessing the number of individuals. However, a single qPCR can yield a quantification of the amount of DNA^38^,^39^. Furthermore, replicating qPCRs, as it is done in replicative endpoint PCR methods should provide with a more precise quantification of the presence of dreissenid DNA. Thus, using the qPCR protocol described here, we can provide water managers with accurate data on the success of control measures in the early invasion stages. This information is crucial for eradications, since it has been demonstrated that effective control measures should be undertaken as early as possible^4^,^25^,^26^.

The H2B single-copy gene marker does not discriminate between the closely related zebra and quagga mussels due to the low levels of interspecies differentiation of this marker between the two species (see Supplementary Figs S2 and S3). The inability to separate zebra from quagga mussels is not a hindrance to implement the method developed in this study. Both dreissenid species have a related native range, similar life histories and morphology^3^,^40^, and they are currently well expanded in Europe^10^,^15^,^19^. The zebra mussel expansion typically occurs earlier^13^,^14^,^41^ with an invasion lag time five times shorter than the quagga mussel^14^,^42^,^43^. Quagga mussels become established in water bodies where zebra mussels are present^7^,^10^, and zebra mussel populations are gradually replaced by quagga mussels^13^,^43^,^44^ with a complete replacement after nine of more years of coexistence^14^. Thus, the lack of discrimination between these two species and the possible incipient quagga mussel specimens in the area studied is not a critical problem to implement the developed method, since regardless which of the two species are present the impact of the invasion is similar^3^,^13^,^14^ or when species occurs concurrently, their effect is even greater^14^. Interestingly, as the first reported presence of the zebra mussel in the Iberian Peninsula was in 2001, we could suspect that the quagga mussel is already present in the Iberian Peninsula. However, the morphological analysis of more than 4,000 adult individuals from two Iberian locations (in the first and the last locations where zebra mussel was detected; see Supplementary Table S1) failed to identify quagga mussel individuals, which may indicate that the invasion of quagga mussel has not yet started or it is in its initial stages.

Additional advantages of our qPCR method developed in this study include: (1) faster results, (2) more cost-effective, (3) and more powerful resolution to detect presence of dreissenid infestation than the previous methods based on visual inspection and/or molecular markers. The traditional microscopic screening methods for veliger larvae detection^25^,^26^ are labour intensive and time-consuming^40^, with a relatively high potential for false positive results. In addition, our method outperforms previous molecular methods aimed at determining only dreissenid DNA presence^4^,^28^,^29^. To the best of our knowledge, this is the first method capable to differentiate specific dreissenid DNA levels from other bivalves in eDNA samples. It should be noted that our method is based on capturing specific environmental DNA from plankton samples including free but also larvae DNA which is a good representation of the larvae movement and propagation^45^.

A goal of this study was to apply our method to actual environmental samples. Five locations were sampled within the recollection program of the Catalan Governmental control and management of zebra mussel expansion^22^ (Table 2). Each location was analysed in two sampling periods as follows: prior to the spawning in spring period; and after the spawning in autumn period^25^. When comparing the visual and molecular analyses results, the molecular analysis provided more positive results than the visual inspection. For instance, neither larvae nor adults were detected by traditional methods in the autumn samples from La Llosa and Boadella Reservoirs as well as in both temporal samples from the Sant Ponç Reservoir. In contrast, the PCR amplifications (subsequently confirmed by Sanger sequencing) were positive in all these samples. These discrepancies between analysis methods may be due to a low number of larvae present in the water column, which is not detected by the microscopical inspection. We should be cautious in the locations where we have failed to detect dreissenid DNA, as there is the possibility that eDNA concentration may vary between adjacent samples^46^,^47^, with the risk of having dreissenid DNA (and thus infestation) in negatively amplified locations. On the contrary, the positive PCR results may indicate an incipient invasion of dreissenid, and these locations should be considered major objectives for the prevention directives by the governmental agencies.

A novel component of our qPCR method is the quantification of the dreissenid infestation level. As a general pattern, we obtained a significantly higher presence of dreissenid DNA in autumn than in spring (inset in Fig. 1). This is in concordance with the vital cycle of zebra mussels, in which major spawning is in summer^14^,^25^ when the water temperature increases above 18 °C^25^. Thus, significantly higher DNA amounts are expected in autumn as a consequence of the higher concentrations of veligers in water samples. This pattern can be observed in four of the five locations analysed (Fig. 1) but only in the Boadella Reservoir the DNA presence was significantly higher in the autumn sample (Fig. 1). La Baells Reservoir presented an opposite pattern with higher DNA presence in the spring season (Fig. 1). The results from this reservoir were in concordance with the Catalan Water Agency (ACA) monitoring plan as it was the only reservoir with a confirmed presence of adult individuals along the year^22^.

One surprising result was the high DNA quantity found in the autumn season for the Gaià Reservoir with no presence of DNA in the spring. A similar situation occurred in La Llosa samples albeit to a lesser extent (Table 2, Fig. 1). ACA only found the presence of larvae but no adults in Gaià in 2012. However, the presence of zebra mussels has not been observed in succeeding years. Based on these results, we suspect that these locations are probably having a flow of dreissenid input, with a high risk of establishment of the invasion in a short term.

In summary, to the best of our knowledge, we have developed the first method for specific detection and quantification of dreissenid DNA in environmental samples based on qPCR. This method outperforms previous methods based on visual and microscopic inspection, and it provides additional information than other molecular methods only based on the detection of the presence of dreissenid DNA. Application of our method allows early detection of dreissenid invasions and fast implementation of control measures.

## Methods

Sequences from zebra mussel were obtained from a previous Massive Parallel Sequencing (MPS) study^11^. De novo assembly generated a total of 2,326 contigs (contig range size = 100–8,697 bp; mean contig size = 457 bp; N50 = 825 bp) and was submitted to the DDBJ/EMBL/GenBank Whole Genome Shotgun project when the contig size was longer than 500 nucleotides under accession number JWHF00000000.

### Stage A: Single-copy marker selection

Homologous sequences of the contig sequences longer than 500 nucleotides were identified using Blast2GO software (version 2.4.2)^48^. The Non-Redundant (NR) NCBI protein database was searched using BlastX with a Cut-Off e-value of 1E-6, Cut-Off length of 30, and 20 Blast Hits. Subsequently, Gene Ontology (GO) terms were assigned according to the Gene Ontology Database with an e-value-Hit-Filter of 1E-6, annotation Cut-Off of 55, and GO Weight of 5. These results were used to predict single-copy genes using homologies with NCBI and Ensembl^49^ databases. The PCR primers for selected genes were designed using Primer3^50^ with default parameters.

#### Adult individual collections for PCR development and validation

The PCR results were validated in three adult zebra mussels from the Aragón Imperial Canal (Ebro River), and the PCR specificity was verified against several mollusc species found in overlapping distribution with zebra mussel: one individual of Spengler’s freshwater mussel (*Margaritifera auricularia*), two Asian clam (*Corbicula fluminea*) and one spike-topped apple snail (*Pomacea sp*.). Furthermore, two individuals of quagga mussel sampled in the Netherlands (52°42′N, 05°18′E) were also included. Whole bodies without shell were preserved in 70% ethanol until processed. DNA isolations were performed using the EZNA Mollusk DNA kit (Omega Bio-Tek), and DNA was eluted in a volume of 200 μl. DNA quality and quantity was verified by agarose gel electrophoresis, Qubit v2.0 fluorometer (Life technologies) and NANODROP spectrophotometer (Thermo Fischer Scientific).

In addition, to infer the possible presence of quagga mussel in waters of the Iberian Peninsula, we collected more than 4,000 dreissenid adult individuals in 2013. All individuals were collected in Ribarroja reservoir (Ebro River; first cited record of the zebra mussel in the Iberian Peninsula^8^; n = 3,013) and La Baells (Llobregat River; last cited record in the Iberian Peninsula^23^; n = 1,230) (see Supplementary Table S1).

#### Tissue PCR amplification

**Figure 2:**
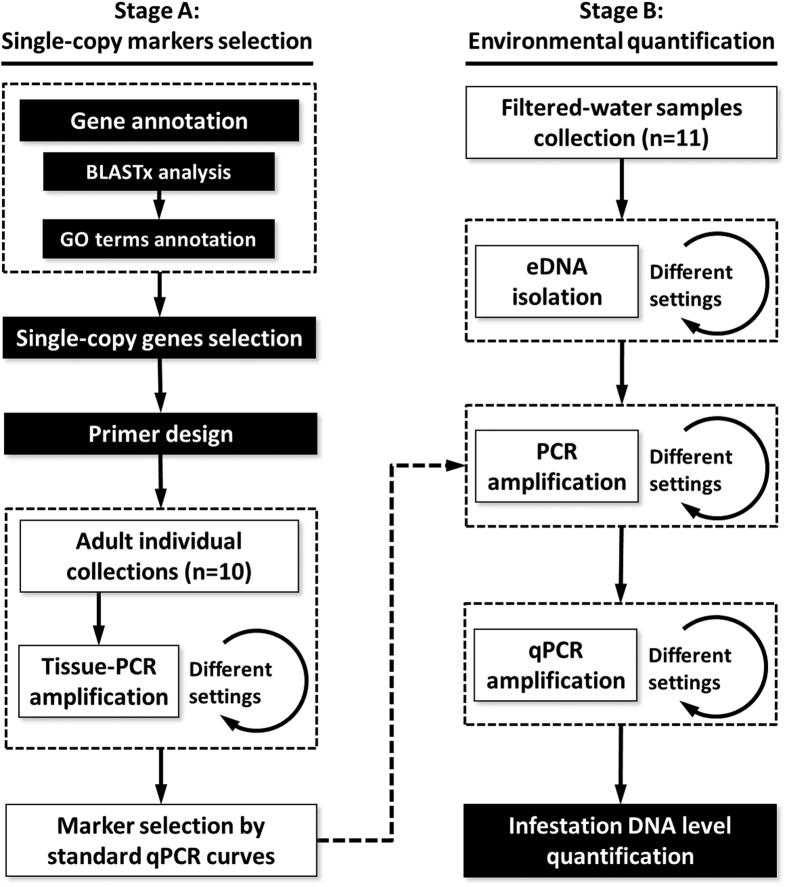
Workflow of experimental design. Black steps: bioinformatic analysis, white steps: laboratory procedures.

All primer sets were tested by end-point PCR in adult tissue DNA using a 2720 Thermal Cycler (Applied Biosystems). PCR assays were set up in 30 μL reactions containing 25–100 ng of genomic DNA, 1X Buffer, 1.5 μM MgCl_2_, 0.8 mM dNTPs, 0.2 μM of each primer, 2.5E-2 u/μL Taq polymerase (BIOLINE) and 3 μL of genomic DNA (approximately 100 ng). Thermal cycles consisted: an initial denaturing step of 3 min at 94 °C; 35 cycles of denaturing at 94 °C for 30 s, annealing at 50 °C for 90 s, and extension at 72 °C for 90 s; and a final extension period of 5 min at 72 °C. The PCR annealing temperature was increased to 60 °C if unspecific results were obtained. Negative controls were included in all PCR runs to ascertain the lack of cross-contamination. The PCR results were verified using 1.5% agarose gel electrophoresis. Finally, the primer pairs that produced a single, clean amplicon were selected for subsequent steps (Fig. 2).

#### Marker selection by standard qPCR curve amplification

The PCR reactions of single-copy genes that demonstrated specificity for dreissenids (zebra and quagga mussels) were used to generate standard curves for the qPCR using an ABI 7300 Real-Time PCR System (Applied Biosystems). First, DNA stock concentrations were normalized to 50 ng/μL and were mixed in a final volume of 200 μL with 100 ng/μL salmon sperm DNA (Invitrogen) as a DNA carrier to minimize loss of zebra mussel DNA in the aliquots. The standard curve was constructed by five consecutive 10-fold dilutions (range of dilutions from 50 to 5E-3 ng/μL). The quantitative qPCR mix was prepared in 20 μL volume reactions with 2 μL of each DNA dilution, 1× SyBR^®^ Green PCR Master Mix (Applied Biosystems) and 0.20 μM of each primer. The amplification temperature profile consisted of an initial step at 50 °C for 10 min; 40 cycles of 95 °C for 15 s and annealing at 60 °C for 1 min; and a dissociation curve consisting of 95 °C for 15 s, 60 °C for 30 s and 95 °C for 15 s. Negative controls were also included to ascertain the lack of cross-contaminations. All samples were tested in triplicate, and the coefficient of variation (CV) was calculated in the triplicates of cycle threshold (CT) to determine the absence of technical and manipulation errors.

The quantification level (QL), defined as the minimum of quantifiable DNA in the standard curve, was determined as the lowest DNA concentration with positive amplification and a CV lower than 0.3. The detection level (DL) was determined as the lower concentration with positive amplification but a CV larger than 0.3. The DL indicates DNA presence but no reliability in the quantification. The dynamic range of the standard curve for every single-copy gene marker was determined using DNA concentrations up to QL and linear correlation. The marker producing the best efficiency and linearity values (lowest CV, QL and DL) was the one selected for dreissenid DNA quantification in the environmental samples. Positive PCR amplifications were directly sequenced by Sanger sequencing to reconfirm identity.

### Stage B: Environmental quantification of dreissenid DNA presence

#### Filtered water sample collection

Samples were obtained from five representative locations (See Table 2 and Supplementary Fig. S4) of different reservoirs in the Northeast Iberian Peninsula following the sampling protocol and procedure of the control and management of the zebra mussel expansion plan^22^. In addition, a sample from Banyoles Lake (Girona, Spain) with no potential risk of zebra mussel invasion due to its isolated geographical distribution was used as a negative control. The presence of adults and larvae in these locations has been tested periodically^22^ using the official Spanish Government procedures^26^. The positive presence of larvae and adult individuals was only detected in La Baells Reservoir since 2011, and ACA also detected larvae but no adults in Gaià Reservoir only in 2012^22^. The remaining three reservoirs were visually negative for the presence of both adults and larvae (See summary of presence of larvae and/or adults in Table 2). For quantitative analysis, two 100 L water samples were collected in spring (before spawning) and two more samples in autumn (after spawning) (Table 2). All of the environmental samples were filtered using a 50 μm diameter mesh to target veliger larvae of *Dreissena* species, rehydrated with water and stored at −20 °C until analysis.

#### Environmental DNA (eDNA) isolation

One of the main points of this method is optimizing the eDNA extraction for all 6 locations (21 water samples) (Table 2) obtaining the most DNA but the lowest presence of PCR inhibitors. After several attempts, the best method resulted using the DNA isolation kit for environmental samples called the FastDNA^TM^ SPIN Kit for Soil (MP Biomedicals). All DNA isolations were validated by 1% agarose gel electrophoresis and quantified using a QUBIT v2.0 fluorometer.

#### PCR amplification and Sanger sequencing validation

The eDNA samples were validated by endpoint PCR using the best molecular marker for dreissenid specificity previously selected in Stage A. Gradient PCR with an annealing temperature ranging from 60 to 70 °C and a Touch-Down PCR with decreasing annealing temperature from 70 to 60 °C (−1 °C/cycle in the first ten cycles) were performed in parallel to optimize PCR amplification. The PCR products were also sequenced using the Sanger method for validation.

#### Quantification of infestation

The dreissenid DNA from the 21 water (6 locations) samples was quantified by qPCR in three consecutive 10-fold dilutions in triplicate. To infer the effect of inhibitors in the eDNA samples, a second qPCR analysis was performed with controlled contaminations (Spikes) using the same dilutions of environmental samples but with the addition of 2 μL of 2 ng/μL zebra mussel DNA extracted from adult tissue. In all cases, the qPCR mix and thermal cycles were performed following Stage A conditions. The quantification results were analysed using 7300 SDS v1.3.1 Software (Applied Biosystems). For positive amplification samples with no inhibitors, concentrations were determined using the standard curve previously developed. The values are presented as the mean concentration (ng/L) with the corresponding standard error of the mean (SEM). A qPCR amplification was considered positive when the following conditions were met: (1) at least one of the dilutions is in the dynamic range of the standard curve and has a cycle threshold (CT) at least six cycles earlier than the no template control; (2) a proportional correspondence among decimal template dilutions and CT amplifications; (3) replicates should present a coefficient of variation lower than 0.3; and (4) specificity must be verified by a single melting peak. Finally, we calculated the estimated number of target gene copies per microliter in positive amplified samples using Avogadro constant times the ratio between DNA concentration (ng/L) and molecular weight of the PCR amplicon.

The data resulting from the quantification was tested for normality by a Kolmogorov-Smirnov test, and values were transformed when necessary (square root transformation for comparisons among the seasons). The statistical analyses for concentration comparisons were performed using the mean value of the two replicates collected in each lake-season combination. These values were compared using two-tailed Student *t*-tests or ANOVA tests with Bonferroni post-hoc correction. All statistical analyses were computed using the IBM SPSS Statistics package (v. 20.0; IBM Corp., USA).

## Additional Information

**How to cite this article**: Peñarrubia, L. *et al*. Validated methodology for quantifying infestation levels of dreissenid mussels in environmental DNA (eDNA) samples. *Sci. Rep.* **6**, 39067; doi: 10.1038/srep39067 (2016).

**Publisher's note:** Springer Nature remains neutral with regard to jurisdictional claims in published maps and institutional affiliations.

## Supplementary Material

Supplementary Information

Supplementary Table S1

## Figures and Tables

**Table 1:** Markers developed for dreissenid identification derived from MPS output and qPCR conduction.

Single-copy gene calling step			PCR amplification step			qPCR amplification step						GenBank accession
MPS Contig reference	BlastX E- value	Predicted gene	Primer sequences (5′ → 3′)	Size (bp)	Ta (°C)	Tm (°C)	E	CV	*R*^2^	DL (ng/μl)	QL (ng/μl)	
Contig000070	3.77E-88*	H3	F: GGTGACACGCTTGGCGTGGA	229	60	—	—	—	—	—	—	JWHF01000070
			R: GCCAGGAACCGTCGCCCTTC									
Contig000076	5.31E-54*	H2B	F: CGCGCGCTCCACTGACAAGA	251	60	85.4692 ± 0.2057	1.9729	0.1541	0.9976	5E-4	5E-4	JWHF01000076
			R: CACCAGGCAGCAGGAGACGC									
Contig000102	1.08E-27	H1	F: TCTTGGCGCCCGCCTTCTTG	214	60	86.0933 ± 0.2612	1.9700	0.2283	0.9954	5E-3	5E-3	JWHF01000102
			R: GTCAGTGCCGTCAACGCCCA									
Contig000913	7.52E-23	MARS	F: AGTCCTCCCAGATTAGCCTGTGC	277	65	80.8778 ± 2.6423	1.9102	0.1772	0.9940	5E-2	5E-1	JWHF01000913
			R: AGATGTCGCGGTGGAGGGCT									

MPS contig reference, Blast Result, predicted gene, Forward (F) and Reverse (R) primer sequences, amplicon size in base pairs, and optimum annealing temperature (Ta) for PCR amplification, Melting temperature (Tm) in Real Time PCR, efficiency (E), coefficient of variation (CV), coefficient of regression (*R*^2^), detection (DL) and quantification (QL) levels and GenBank accession number for four single-copy predicted genes selected for zebra mussel. *Significant BlastX E-value <1E-50.

**Table 2:** Location, coordinates, sampling date (S = spring, A = autumn), zebra mussel presence previously diagnosed by ACA (Catalan Water Agency), and molecular diagnostic results (+ = positive; ND = non detected) for all samples analyzed.

Location, reservoir	Sample acquisitions	Sampling Date	Visual diagnostic by ACA			Molecular diagnostic	
	Latitude/Longitude		larvae	adults	PCR	qPCR (ng/L ± SEM)	Target copies/μL ( ± SEM)
La Baells	42°08′N/01°54′E	S: 08/05/2014	+	+	+	19.935 ± 6.743	7.25E + 4 ± 2.45E + 4
		A: 01/10/2014	+	+	+	5.481 ± 0.411	1.99E + 4 ± 1.49E + 3
Gaià	41°11′N/01°19′E	S: 04/06/2014	ND	ND	ND	ND	ND
		A: 01/10/2014	ND	ND	+	182.900 ± 11.300	6.65E + 5 ± 4.11E + 4
La Llosa	42°05′N/01°34′E	S: 21/05/2014	ND	ND	ND	ND	ND
		A: 29/09/2014	ND	ND	+	26.745 ± 2.615	9.72E + 4 ± 9.51E + 3
Sant Ponç	41°57′N/01°36′E	S: 21/05/2014	ND	ND	+	6.580 ± 1.313	2.39E + 4 ± 4.77E + 3
		A: 29/09/2014	ND	ND	+	14.368 ± 4.160	5.22E + 4 ± 1.51E + 4
Boadella	42°20′N/02°50′E	S: 14/05/2014	ND	ND	ND	3.938 ± 0.518	1.43E + 4 ± 1.88E + 3
		A: 17/09/2014	ND	ND	+	60.144 ± 6.096	2.19E + 5 ± 2.22E + 4
Banyoles Lake	42°07′N/02°45′E	26/11/2013	ND	ND	ND	ND	ND

SEM: standard error of the mean.
