# Supplementary material for: Validated methodology for quantifying infestation levels of dreissenid mussels in environmental DNA (eDNA) samples
Source: Sci Rep. 2016 Dec 14;6:39067. doi: 10.1038/srep39067 (PMC5155230; doi:10.1038/srep39067)
Supplement: Supplementary Information [file srep39067-s1.pdf]

Validated methodology for quantifying infestation levels of dreissenid mussels in environmental DNA (eDNA) samples.

Luis Peñarrubia, Carles Alcaraz, Abraham bij de Vaate, Nuria Sanz, Carles Pla, Oriol Vidal, Jordi Viñas.

Supplementary Information

Supplementary Figure S1. Gene Ontology (GO) genes annotation for MPS sequences

Supplementary Figure S2. Agarose gel (1.5%) of representative adult individuals in the species specificity PCR. DP: *D. polymorpha*, DR: *D. rostriformis*, CF: *C. fluminea*, PS: *Pomacea sp*., MA: *M. auricularia*, Neg: negative PCR control.

Supplementary Figure S3. Alignment of H2B sequences with the MPS sequence of reference. DP adult tissue represents all H2B sequences for the three *D. polymorpha* adult individuals. DR adult tissue represents H2B sequences for the two *D. rostriformis* adult individuals analyzed. Water samples sequence represents all environmental samples, all of them matching completely with adult *D. polymorpha* H2B sequences.

Supplementary Figure S4. Map of the situation of the water samples analyzed in this study. All locations are situated in the Northwest Iberian Peninsula (grey area). The map was generated by the authors using the vector drawing program *Inkscape version 0.48.1* (https://inkscape.org/en/).

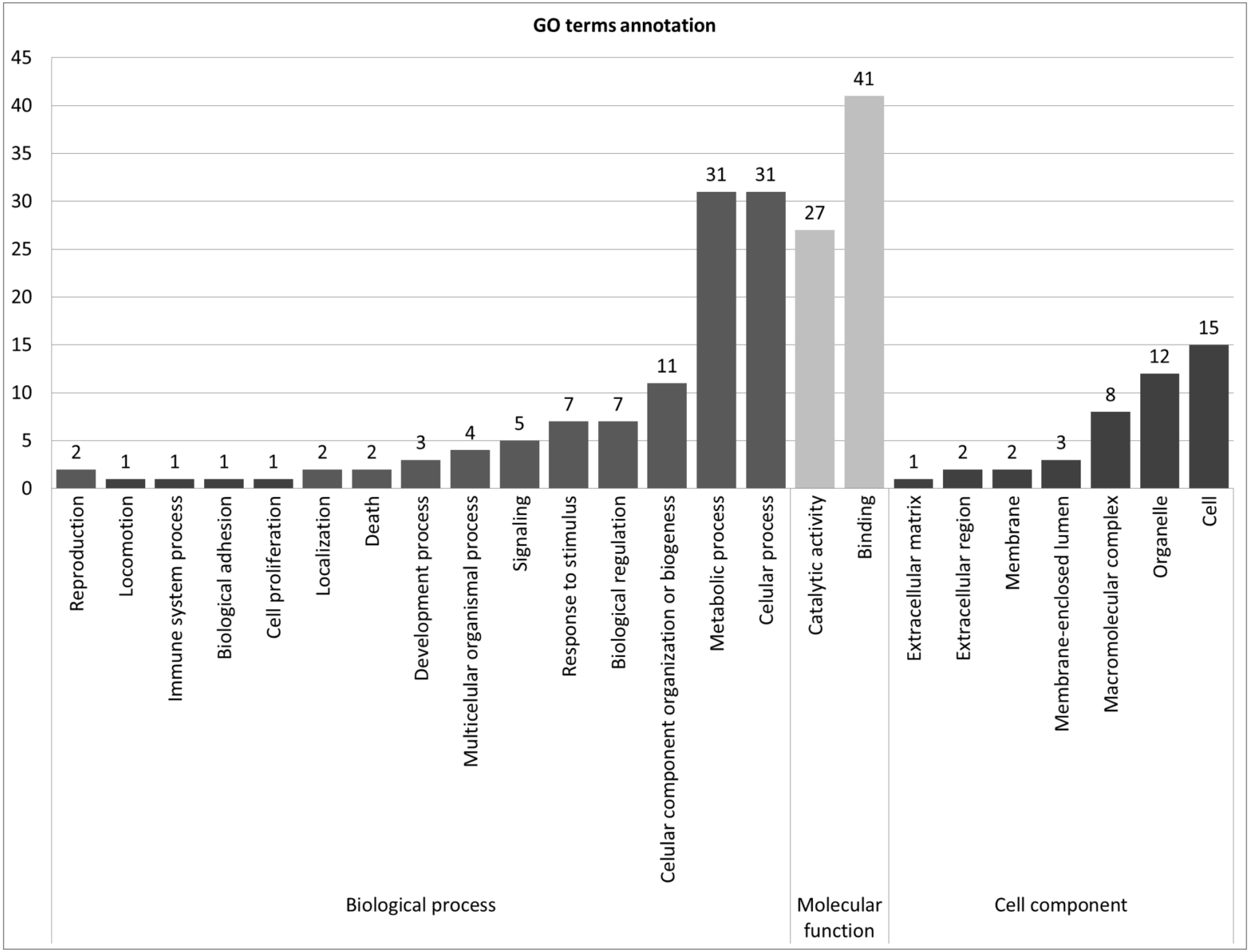

Supplementary Figure S1

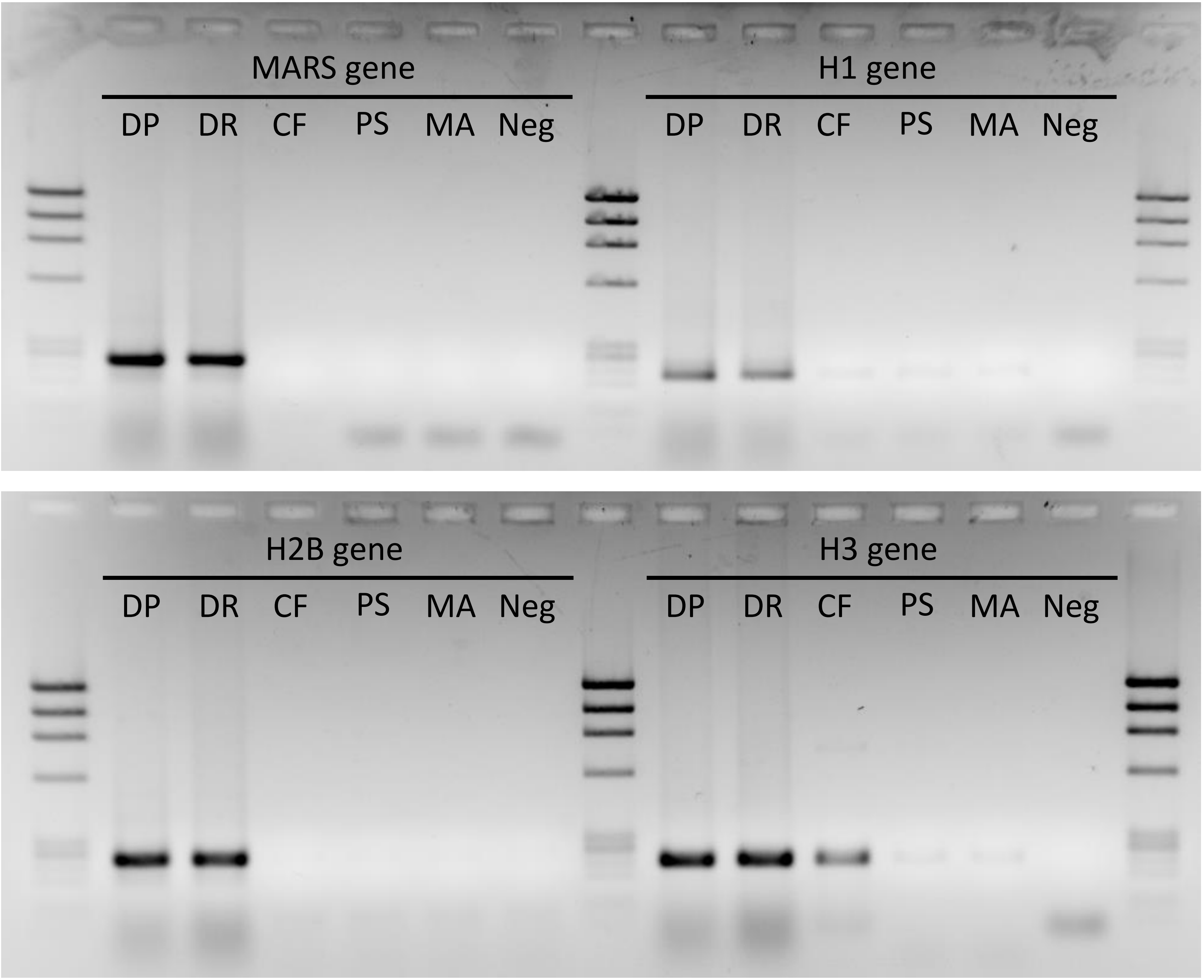

Supplementary Figure S2

```
                 1   5          15         25         35         45         55
                 |   |          |          |          |          |          |
Contig000076     CGCGCGCTCC ACTGACAAGA AGAAGCGCAG GAGGAGGAGG GAATCCTACG CCATCTACAT
DP adult tissue  .......... .......... .......... .......... .......... ..........
DR adult tissue  .......... .......... .......... .......... .....A.... ..........
Water samples    .......... .......... .......... .......... .......... ..........

                     65         75         85         95        105        115
                     |          |          |          |          |          |
Contig000076     CTACAAAGTC TTGAAGCAGG TGCACCCCGA CACCGGAGTG TCCAGCAAGG CCATGTCGAT
DP adult tissue  .......... .......... .......... .......... .......... ..........
DR adult tissue  .......... C.C....... .......... .......... .......... .......C..
Water samples    .......... .......... .......... .......... .......... ..........

                     125        135        145        155        165        175
                     |          |          |          |          |          |
Contig000076     CATGAACAGC TTTGTCAACG ACATCTTCGA GCGCATTGCT GCCGAGGCTT CCCGCCTTGC
DP adult tissue  .......... .......... .......... .......... .......... ..........
DR adult tissue  .......... .......... .......... ......C... .......... ..........
Water samples    .......... .......... .......... .......... .......... ..........

                     185        195        205        215        225        235
                     |          |          |          |          |          |
Contig000076     CCACTACAAC AAGCGATCCA CCATCACAAG CAGAGAGATC CAGACCGCTG TGCGTCTCCT
DP adult tissue  .......... .......... .......... .......... .......... ..........
DR adult tissue  .......... .......... .......... .......... .......... ..........
Water samples    .......... .......... .......... .......... .......... ..........

                     245    251
                     |      |
Contig000076     GCTGCCTGGT G
DP adult tissue  .......... .
DR adult tissue  .......... .
Water samples    .......... .
```

Supplementary Figure S3

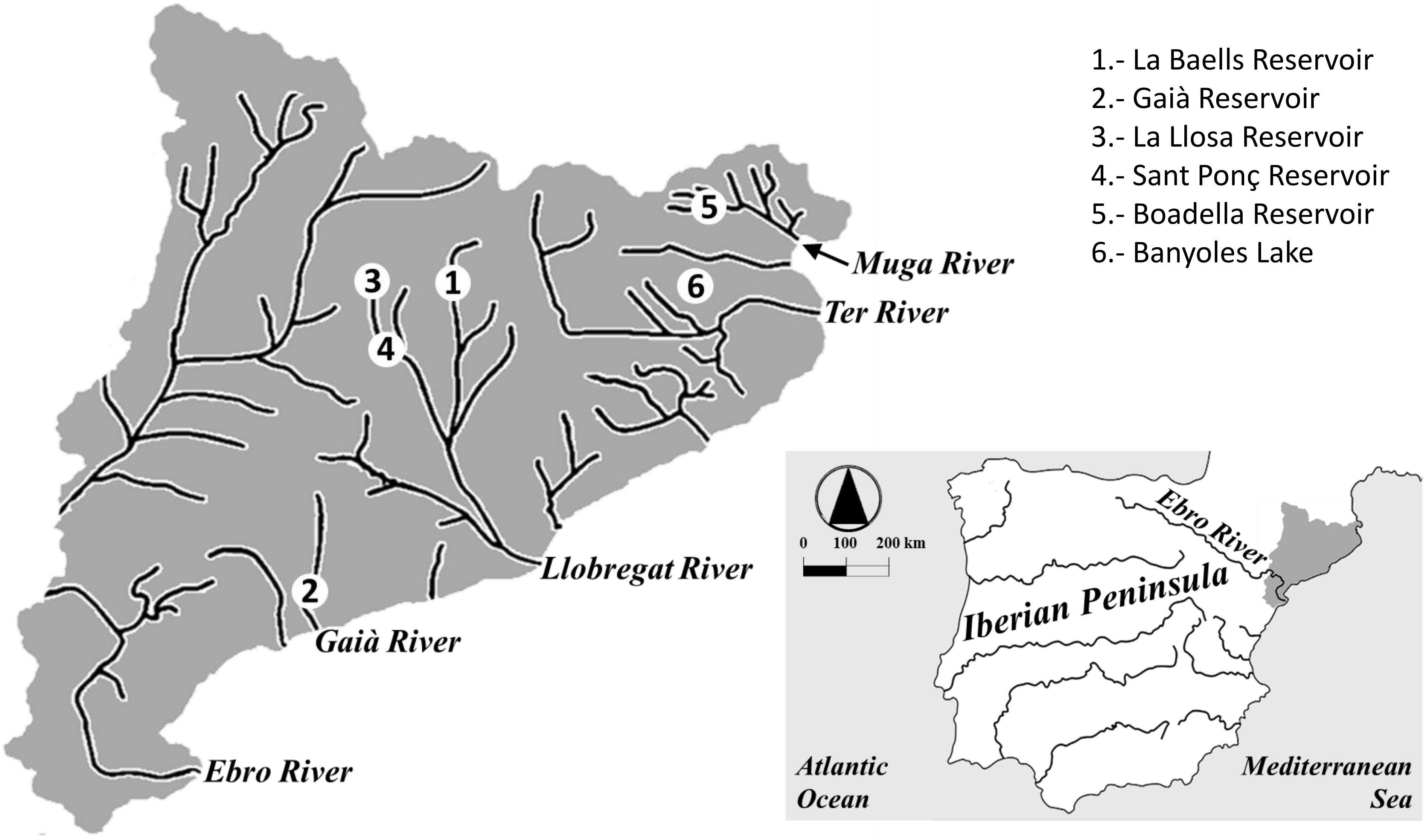

Supplementary Figure S4
